# 移植预处理强度对骨髓增生异常肿瘤异基因造血干细胞移植结局的影响：多中心回顾性队列研究

**DOI:** 10.3760/cma.j.cn121090-20251029-00490

**Published:** 2026-03

**Authors:** 铃 王, 子璐 张, 若璇 张, 红如 陈, 敏利 胡, 妍敏 赵, 威 石, 阳 曹, 晓霞 胡

**Affiliations:** 1 上海交通大学医学院附属瑞金医院转化医学中心，上海 200025 National Research Center for Translational Medicine, Ruijin Hospital, Shanghai Jiao Tong University School of Medicine, Shanghai 200025, China; 2 东南大学附属南通市第一人民医院血液科，南通 226001 Department of Hematology, Southeast University Affiliated Nantong First People's Hospital, Nantong 226001, China; 3 华中科技大学同济医学院附属同济医院血液科，武汉 430033 Department of Hematology, Tongji Hospital, Tongji Medical College, Huazhong University of Science and Technology, Wuhan 430033, China; 4 华中科技大学同济医学院附属协和医院血液科，武汉 430000 Department of Hematology, Union Hospital, Tongji Medical College, Huazhong University of Science and Technology, Wuhan 430000, China; 5 浙江大学医学院附属第一医院骨髓移植中心，杭州 310003 Bone Marrow Transplantation Center, the First Affiliated Hospital, Zhejiang University School of Medicine, Hangzhou 310003, China; 6 浙江省台州医院血液科，台州 317000 Department of Hematology, Taizhou Hospital of Zhejiang Province, Taizhou 317000, China

**Keywords:** 骨髓增生异常肿瘤, 异基因造血干细胞移植, 预处理强度, TCI评分, 非复发死亡, Myelodysplastic neoplasms, Allogeneic hematopoietic stem cell transplantation, Conditioning intensity, TCI score, Non-relapse mortality

## Abstract

**目的:**

探讨不同移植预处理强度（TCI）对骨髓增生异常肿瘤（MDS）患者异基因造血干细胞移植（allo-HSCT）结局的影响。

**方法:**

本研究为多中心回顾性队列研究，纳入2019年2月至2025年4月国内4家移植中心接受allo-HSCT的337例MDS患者。依据TCI评分将预处理强度分为TCI-1（1～2分，低强度）、TCI-2（2.5～3.5分，中等强度）、TCI-3（4～6分，高强度）三组。采用Fine-Gray竞争风险模型分析非复发死亡和复发，Kaplan-Meier法计算总生存（OS）率，并用Log-rank进行比较。为消除基线混杂偏倚，采用逆概率加权法（IPTW）均衡组间协变量；在此基础上，进一步采用双重稳健模型（DR）结合Fine-Gray竞争风险模型，评估TCI对非复发死亡的独立影响。

**结果:**

未加权分析显示，TCI-1、TCI-2和TCI-3三组移植后3年非复发死亡率（NRM）分别为7.7％（95％ *CI*：0.4％～15.1％）、8.3％（95％ *CI*：4.3％～12.3％）和27.1％（95％ *CI*：18.0％～36.3％）（*P*＝0.008）。TCI-1和TCI-2两组发生非复发死亡的风险之间差异无统计学意义（*sHR*＝1.106，95％ *CI*：0.371～3.298，*P*＝0.857），TCI-3组发生非复发死亡的风险显著高于TCI-1组（*sHR*＝4.023，95％ *CI*：1.414～11.452，*P*＝0.009）和TCI-2组（*sHR*＝3.673，95％*CI*：1.951～6.912，*P*<0.001）。多因素分析显示TCI-3、年龄≥50岁和造血干细胞移植合并症指数（HCT-CI）评分>2分是非复发死亡的独立危险因素。经IPTW校正后，结果保持一致：TCI-3组患者的非复发死亡风险仍显著高于TCI-1组（*sHR*＝6.090）和TCI-2组（*sHR*＝4.562）。进一步采用DR校正潜在残余混杂后，TCI-3仍是非复发死亡的独立危险因素（*sHR*＝5.011，95％*CI*：1.118～22.452，*P*＝0.035）。比较不同时间点各组非复发死亡对移植失败的贡献率，随着预处理强度增加，非复发死亡对移植失败的贡献率呈明显上升趋势，尤其在TCI-3组中表现最为突出。移植后3年非复发死亡对移植失败的贡献率为39.0％。Kaplan-Meier曲线显示TCI-3组3年OS率最低（61.2％，95％ *CI*：51.5％～72.7％）。三组间3年累积复发率差异无统计学意义。

**结论:**

高强度预处理未能转化为复发率的下降，反而显著增加allo-HSCT后非复发死亡风险并损害OS。低、中强度预处理能在控制毒性与维持疗效之间取得较优平衡。

## Abstract (en)

**Objective:**

To investigate the effect of transplant conditioning intensity（TCI）on transplant outcomes in patients with myelodysplastic neoplasms（MDS）undergoing allogeneic hematopoietic stem cell transplantation（allo-HSCT）.

**Methods:**

This multicenter retrospective cohort study included 337 patients with MDS undergoing allo-HSCT between February 2019 and April 2025 in four transplant centers in China. Patients were categorized based on TCI scores into TCI-1（1–2 points, low intensity）, TCI-2（2.5–3.5 points, moderate intensity）, and TCI-3（4–6 points, high intensity）groups. The Fine-Gray competing risk model was applied to analyze nonrelapse mortality（NRM）and relapse. Overall survival（OS）was estimated employing the Kaplan-Meier method and compared using the log-rank test. Inverse probability of treatment weighting（IPTW）was performed to balance covariates among groups to eliminate baseline confounding bias. Furthermore, a doubly robust（DR）model combined with the Fine-Gray competing risk model was applied to assess the independent effect of TCI on NRM.

**Results:**

Unweighted analysis revealed that the 3-year NRM post-transplantation in the TCI-1, TCI-2, and TCI-3 groups was 7.7％（95％ *CI*: 0.4％–15.1％）, 8.3％（95％ *CI*: 4.3％–12.3％）, and 27.1％（95％ *CI*: 18.0％–36.3％）, respectively（*P*＝0.008）. No significant difference in the risk of NRM was observed between the TCI-1 and TCI-2 groups（*sHR*＝1.106, 95％ *CI*: 0.371–3.298, *P*＝0.857）, whereas the risk of NRM in the TCI-3 group was significantly higher than that in the TCI-1（*sHR*＝4.023, 95％ *CI*: 1.414–11.452, *P*＝0.009）and TCI-2 groups（*sHR*＝3.673, 95％ *CI*: 1.951–6.912, *P*<0.001）. The multivariable analysis identified TCI-3, age of ≥50 years, and hematopoietic cell transplantation-comorbidity index score of >2 points as independent risk factors for NRM. After IPTW adjustment, the results remained consistent: the risk of NRM in the TCI-3 group remained significantly higher than that in the TCI-1（*sHR*＝6.090）and TCI-2 groups（*sHR*＝4.562）. The comparison of the contribution of NRM to transplant failure across groups at different time points revealed a significantly increased contribution of NRM to transplant failure with increasing conditioning intensity, especially in the TCI-3 group（*sHR*＝5.011, 95％ *CI*: 1.118–22.452, *P*＝0.035）. NRM within 3 years posttransplantation accounted for 39.0％ of transplant failures. The Kaplan-Meier curve revealed that the 3-year OS rate was the lowest in the TCI-3 group（61.2％, 95％ *CI*: 51.5％–72.7％）. No statistically significant difference in the 3-year cumulative incidence of relapse was observed across the three groups.

**Conclusion:**

High-intensity conditioning failed to translate into a reduction in relapse rate but rather significantly increased the risk of NRM and compromised OS after allo-HSCT. Low- and moderate-intensity conditioning achieves a better balance between controlling toxicity and maintaining efficacy.

骨髓增生异常肿瘤（MDS）是一组起源于造血干/祖细胞的异质性克隆性疾病，其特征为骨髓细胞发育异常、外周血一系或多系血细胞减少以及急性髓系白血病（AML）转化风险增加[1]–[2]。异基因造血干细胞移植（allo-HSCT）是目前唯一治愈MDS的方法[3]。MDS患者首次诊断时多数已超过60岁，欧美新发MDS患者中位诊断年龄为73～76岁，我国目前尚未有完整MDS流行病学资料，上海地区2004年至2007年的调查发现，MDS中位发病年龄为62岁[4]–[5]。高龄患者群体常伴随多种合并症，对预处理方案的耐受性降低，移植相关死亡风险高[6]。

MDS患者的最佳预处理强度仍有争议。相比于减低强度预处理（RIC），清髓性预处理（MAC）虽可降低复发风险，但其毒性较大，显著增加早期非复发死亡率（NRM）[7]。欧洲血液与骨髓移植学会（EBMT）Ⅲ期随机临床试验表明，MAC与RIC在移植后非复发死亡、复发及长期生存方面无显著差异，MAC早期死亡风险略高，并未显著改善生存结局[8]。而BMT CTN 0901临床研究中，MAC组复发率低，显示出生存获益[7]。提示传统MAC/RIC二分法难以全面、准确反映预处理强度对MDS患者移植结局的真实影响。2020年EBMT提出量化移植预处理强度（TCI），为预处理方案强度评估提供了新工具[9]。TCI评分体系有效预测AML患者不同预处理强度早期非复发死亡及复发风险[10]。MDS患者与AML在疾病生物学特征、年龄结构及治疗背景方面存在显著差异，AML中建立的TCI评分体系是否适用于MDS人群，尚缺乏系统性研究。鉴于此，本研究依托国内4家大型移植中心的临床数据开展多中心回顾性队列分析，旨在探讨基于TCI的不同预处理强度对MDS患者移植结局的影响，以期为临床个体化预处理强度选择提供依据。

## 病例与方法

1. 病例资料：本研究为回顾性队列研究，纳入2019年2月至2025年4月在上海交通大学医学院附属瑞金医院、浙江大学医学院附属第一医院、华中科技大学同济医学院附属同济医院及华中科技大学同济医学院附属协和医院4家移植中心首次接受allo-HSCT的337例MDS患者，所有患者诊断均符合WHO 2022 MDS诊断标准，并排除合并其他血液系统恶性肿瘤。随访截止日期为2025年7月26日，中位随访923（3～2 254）d。

2. 预处理方案及TCI分组：根据患者的年龄、体能状态和合并症选择预处理方案。MAC主要包括以白消安（Bu）联合环磷酰胺（Cy）为基础的方案（Bu：3.2 mg·kg^−1^·d^−1^，静脉滴注，持续4 d；Cy：60 mg·kg^−1^·d^−1^，静脉滴注，持续2 d）或Bu联合氟达拉滨（Flu）的方案（Bu：3.2 mg·kg^−1^·d^−1^，连续静脉滴注，持续3～4 d；Flu：30 mg·m^−2^·d^−1^，静脉滴注，持续5 d）；RIC主要包括FluBu2（Flu：30 mg·m^−2^·d^−1^，静脉滴注，持续5 d；Bu：3.2 mg·kg^−1^·d^−1^，静脉滴注，持续2 d）或美法仑（Mel）联合Flu（Mel：100 mg/m^2^，静脉滴注，1 d；Flu：30 mg·m^−2^·d^−1^，静脉滴注，持续5 d）。参考Spyridonidis等提出的TCI评分体系，本研究根据预处理方案中各药物种类及对应剂量水平赋予相应权重分值，将所有药物权重分值相加得到患者TCI总分，同时结合MDS患者的疾病特点与移植预处理临床实践，未对原评分体系的药物权重、剂量分级及评分区间进行调整，据此分为低强度TCI-1（1～2分）、中等强度TCI-2（2.5～3.5分）、高强度TCI-3（4～6分）[9]。

3. GVHD及感染预防：移植物抗宿主病（graft-versus-host disease，GVHD）预防以环孢素A+霉酚酸酯+小剂量甲氨蝶呤为主[11]–[12]。亲缘单倍体供者（HID）和无关全合供者（MUD）加用7.5 mg/kg抗胸腺细胞球蛋白（ranti-thymocyte globulin，ATG）或80～100 mg/kg后置环磷酰胺（posttransplant cyclophosphamide，PT-Cy）。阿昔洛韦及抗真菌药物预防病毒和真菌感染，药物选择及疗程见文献[13]–[14]。来特莫韦预防高危患者巨细胞病毒（cytomegalovirus，CMV）感染[14]–[15]。

4. 统计学处理：采用R 4.5.1进行统计学分析及数据可视化，主要使用cmprsk、survival、survey和mstate等软件包。连续变量以中位数（*IQR*）表示，组间比较采用Kruskal-Wallis *H*检验。分类变量以例数（构成比）表示，组间比较采用*χ*^2^检验或Fisher精确概率法。

研究的主要结局指标包括总生存（OS）、非复发死亡及复发。OS定义为自移植日（day 0）起至任意原因死亡的时间，存活至末次随访者予以删失。非复发死亡定义为因MDS复发或进展以外原因所致的死亡，以复发为竞争事件。复发定义为移植后MDS的复发或进展，以非复发死亡为竞争事件，末次随访时未复发者予以删失。

采用Fine-Gray竞争风险模型估计NRM和累积复发率，并用Gray检验比较组间差异；采用Kaplan-Meier法计算OS，Log-rank检验进行组间差异比较。为探讨影响非复发死亡的独立危险因素，首先对各临床变量进行单因素分析，将在单因素分析中*P*<0.10的变量纳入多因素Fine-Gray回归模型，结果以亚分布风险比（*sHR*）及95％置信区间（*CI*）表示。针对基线协变量分布不平衡的情况，采用逆概率加权（IPTW）及双重稳健（DR）的Fine-Gray回归模型进行校正：DR模型通过在IPTW加权基础上进一步纳入全部基线协变量实现。加权后重新对主要结局指标进行分析。基于mstate多状态模型构建了从“移植后→非复发死亡或复发”的转移矩阵，计算非复发死亡在移植失败中的贡献率。双侧*P*<0.05为差异有统计学意义，两两比较时检验水准按Bonferroni法校正，校正后*P*<0.017为差异有统计学意义。

## 结果

1. 基线特征：患者中位年龄49（*IQR*：38～57）岁，其中46.0％（155/337）患者年龄≥ 50岁。TCI-1组占16.3％（55/337），TCI-2组占56.1％（189/337），TCI-3组占27.6％（93/337）。队列以MDS-低原始细胞（LB）亚型为主（55.5％，187/337），其次为原始细胞增多1（IB1）（20.8％，70/337）和原始细胞增多2（IB2）（10.7％，36/337）亚型。各亚型在不同TCI组间的分布差异无统计学意义（*P*＝0.157）。年龄、GVHD预防方式、供者类型及移植物中单个核细胞（MNC）计数在不同TCI组间差异具有统计学意义（表1）。

**表1:** 不同移植预处理强度（TCI）分组骨髓增生异常肿瘤（MDS）患者基线临床特征比较

临床特征	TCI-1组 （55例）	TCI-2组 （189例）	TCI-3组 （93例）	*P*值
年龄［*M*（*IQR*）］	56（46～60）	49（37.5～57）	45（36.5～52）	<0.001
性别［例（％）］				0.226
男	34（61.8）	92（48.7）	47（50.5）	
女	21（38.2）	97（51.3）	46（49.5）	
MDS亚型［例（％）］				0.157
LB	31（56.4）	102（54.0）	54（58.1）	
IB1	7（12.7）	47（24.9）	16（17.2）	
IB2	7（12.7）	23（12.2）	6（6.5）	
bi-TP53	3（5.5）	4（2.1）	3（3.2）	
MDS-f	0（0）	0（0）	1（1.1）	
MDS-h	5（9.1）	11（5.8）	11（11.8）	
NA	2（3.6）	2（1.1）	2（2.2）	
IPSS-R［例（％）］				0.694
极低危	2（3.7）	2（1.2）	1（1.2）	
低危	9（16.7）	17（9.9）	10（12.5）	
中危	15（27.8）	54（31.6）	26（32.5）	
高危	10（18.5）	25（14.6）	14（17.5）	
极高危	18（33.3）	73（42.7）	29（36.2）	
NA	1（1.8）	18（9.5）	13（14.0）	
IPSS-M［例（％）］				0.110
极低危	0（0）	3（1.8）	0（0）	
低危	7（14.0）	6（3.7）	6（8.1）	
中低危	7（14.0）	17（10.4）	13（17.6）	
中高危	9（18.0）	24（14.6）	16（21.6）	
高危	11（22.0）	56（34.1）	18（24.3）	
极高危	16（32.0）	58（35.4）	21（28.4）	
NA	5（9.1）	25（13.2）	19（20.4）	
移植前是否经过桥接治疗［例（％）］				0.076
是	22（40.0）	101（53.4）	55（59.1）	
否	33（60.0）	88（46.6）	38（40.9）	
移植前骨髓原始细胞比例［例（％）］				0.149
<5％	37（67.3）	111（58.7）	65（69.9）	
≥5％	18（32.7）	78（41.3）	28（30.1）	
供者类型［例（％）］			0.002
HID	35（63.6）	152（80.4）	54（58.1）	
MSD	10（18.2）	20（10.6）	21（22.6）	
MUD	10（18.2）	17（9.0）	18（19.4）	
HCT-CI［例（％）］				0.106
≤2分	43（78.2）	167（88.4）	83（89.2）	
>2分	12（21.8）	22（11.6）	10（10.8）	
GVHD预防［例（％）］	0.003
ATG	52（94.5）	171（90.5）	72（77.4）	
PT-Cy	0（0）	11（5.8）	14（15.1）	
其他	3（5.5）	7（3.7）	7（7.5）	
移植物中MNC计数［×10^8^/kg，*M*（*IQR*）］	10.51（6.88～14.19）	11.95（8.49～16.00）	9.85（7.42～13.24）	0.030
移植物中CD34^+^细胞计数［×10^6^/kg，*M*（*IQR*）］	5.90（4.42～7.96）	5.97（4.47～7.50）	5.39（4.00～7.30）	0.326

**注** TCI评分1～2分为TCI-1组、2.5～3.5分为TCI-2组、4～6分为TCI-3组。LB：低原始细胞负荷型；IB1：原始细胞增多型1；IB2：原始细胞增多型2；bi-TP53：TP53双等位基因异常型；MDS-f：伴纤维化的骨髓增生异常肿瘤；MDS-h：低增生型骨髓增生异常肿瘤；NA：数据缺失无法评估；IPSS-R：修订版国际预后评分系统；IPSS-M：分子国际预后评分系统；HID：半相合供者；MSD：同胞全合供者；MUD：非血缘全合供者；HCT-CI：造血干细胞移植合并症指数；GVHD：移植物抗宿主病；ATG：抗胸腺细胞球蛋白；PT-Cy：后置环磷酰胺；MNC：单个核细胞

2. TCI对非复发死亡的影响：不同TCI组移植结局见表2。以TCI-1组为参照，TCI-2组在整个随访期间的非复发死亡亚分布风险无明显差异（*sHR*＝1.106，95％*CI*：0.371～3.298，*P*＝0.857）。TCI-3组的非复发死亡的亚分布风险显著高于TCI-1组（*sHR*＝4.023，95％*CI*：1.414～11.452，*P*＝0.009）和TCI-2组（*sHR*＝3.673，95％*CI*：1.951～6.912，*P*<0.001）。在早期时间点（100 d、180 d）亦观察到类似趋势，TCI-3组NRM持续高于TCI-1和TCI-2组。多因素分析显示TCI-3（*sHR*＝5.797，95％*CI*：2.081～16.151，*P*＝0.001）、年龄≥50岁（*sHR*＝1.942，95％*CI*：1.025～3.677，*P*＝0.042），造血干细胞移植合并症指数（HCT-CI）评分>2分（*sHR*＝2.259，95％*CI*：1.093～4.669，*P*＝0.028）是非复发死亡的独立危险因素（表3）。

**表2:** 不同移植预处理强度（TCI）分组骨髓增生异常肿瘤（MDS）患者的移植结局发生率比较［％（95％ *CI*）］

结局事件	TCI-1组（55例）	TCI-2组（189例）	TCI-3组（93例）	*P*值
100 dⅡ～Ⅳ度aGVHD	21.8（10.8～32.9）	12.7（7.9～17.5）	24.7（15.9～33.6）	0.032
100 dⅢ～Ⅳ度aGVHD	7.3（0.3～14.2）	7.9（4.1～11.8）	17.2（9.5～24.9）	0.040
2年轻度cGVHD	38.1（21.7～54.4）	41.2（32.6～49.8）	33.2（21.8～44.6）	0.996
2年中重度cGVHD	19.7（6.8～32.7）	13.4（7.6～19.2）	35.6（23.8～47.3）	<0.001
非复发死亡
100 d	1.8（0～5.4）	3.7（1.0～6.4）	11.8（5.2～18.4）	0.041
180 d	5.6（0～11.9）	6.5（2.9～10.1）	19.4（11.3～27.4）	0.030
1年	7.7（0.4～15.1）	8.3（4.3～12.3）	22.7（14.1～31.2）	0.023
3年	7.7（0.4～15.1）	8.3（4.3～12.3）	27.1（18.0～36.3）	0.008
3年OS	70.3（56.3～87.8）	78.7（72.6～85.3）	61.2（51.5～72.7）	0.010
复发
1年	17.2（6.8～27.6）	14.6（9.4～19.8）	9.8（3.7～15.9）	0.402
3年	21.5（10.0～32.9）	21.5（14.9～28.1）	19.8（10.7～28.8）	0.957

**注** TCI评分1～2分为TCI-1组、2.5～3.5分为TCI-2组、4～6分为TCI-3组。aGVHD：急性移植物抗宿主病；cGVHD：慢性移植物抗宿主病；OS：总生存

**表3:** 影响骨髓增生异常肿瘤（MDS）患者移植后非复发死亡的单因素及多因素分析

变量	单因素分析		多因素分析	
	*sHR*（95％ *CI*）	*P*值	*sHR*（95％ *CI*）	*P*值
年龄				
<50岁	参照	–	–	–
≥50岁	1.947（1.076～3.523）	0.028	1.942（1.025～3.677）	0.042
性别				
女	参照	–	–	–
男	1.478（0.817～2.672）	0.196	–	–
MDS亚型				
LB	参照	–	–	–
IB1	1.178（0.542～2.561）	0.680	–	–
IB2	1.918（0.807～4.555）	0.140	–	–
bi-TP53	未发生事件	–	–	–
MDS-h	2.004（0.824～4.871）	0.130	–	–
供者类型		
MSD	参照	–	参照	–
HID	3.028（0.959～9.560）	0.059	5.169（0.942～8.356）	0.059
MUD	1.695（0.389～7.382）	0.482	2.476（0.379～16.19）	0.344
移植前HCT-CI评分				
≤2分	参照	–	参照	–
>2分	2.073（1.030～4.175）	0.041	2.259（1.093～4.669）	0.028
移植前骨髓原始细胞比例				
<5％	参照	–	–	–
≥5％	1.630（0.910～2.921）	0.101	–	–
移植前是否桥接治疗				
否	参照	–	–	–
是	0.803（0.448～1.439）	0.461		
GVHD预防方式		
ATG	参照	–	–	–
PT-Cy	1.193（0.431～3.304）	0.734	–	–
其他	0.788（0.209～2.965）	0.724	–	–
TCI分组		
TCI-1	参照	–	参照	–
TCI-2	1.097（0.366～3.294）	0.868	1.123（0.379～3.331）	0.834
TCI-3	4.036（1.421～11.462）	0.009	5.797（2.081～16.151）	0.001
移植物中MNC计数	1.024（0.973～1.078）	0.360	–	–
移植物中CD34^+^细胞计数	1.011（0.896～1.140）	0.864	–	–

**注** LB：低原始细胞负荷型；IB1：原始细胞增多型1；IB2：原始细胞增多型2；bi-TP53：TP53双等位基因异常型；MDS-h：低增生型骨髓增生异常肿瘤；MSD：同胞全合供者；HID：半相合供者；MUD：非血缘全合供者；HCT-CI：造血干细胞移植合并症指数；GVHD：移植物抗宿主病；ATG：抗胸腺细胞球蛋白；PT-Cy：后置环磷酰胺；TCI：预处理强度，TCI评分1～2分为TCI-1组、2.5～3.5分为TCI-2组、4～6分为TCI-3组；MNC：单个核细胞；–：无数据

为消除潜在混杂因素影响，我们对年龄、HCT-CI评分、骨髓原始细胞比例、供者类型、GVHD预防方式、是否桥接治疗、移植物中MNC计数以及移植物中CD34^+^细胞计数进行了IPTW。IPTW后TCI对移植后非复发死亡的影响保持不变（图1A、B）。TCI-3组非复发死亡风险显著高于TCI-1组（*sHR*＝6.090，95％*CI*：1.600～23.215，*P*＝0.008）和TCI-2组（*sHR*＝4.562，95％*CI*：2.355～8.838，*P*<0.001）（图1B）。

**图1:**
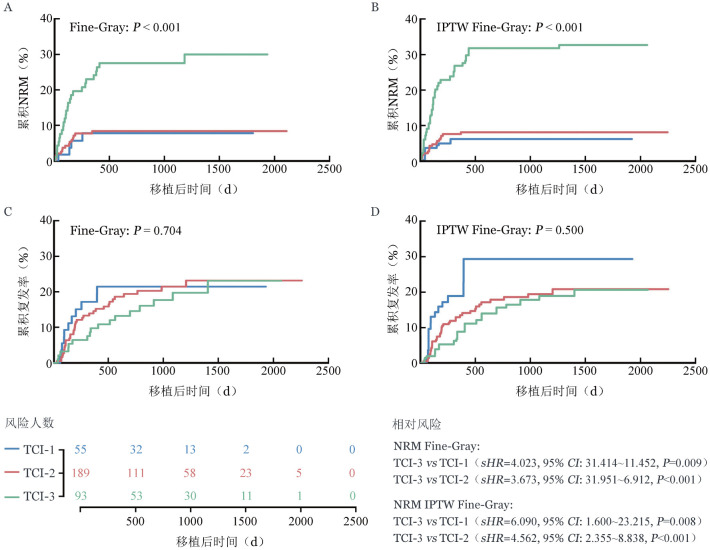
IPTW前后骨髓增生异常肿瘤不同TCI分组累积NRM和累积复发率 **A** 竞争风险非复发死亡；**B** 逆概率加权竞争风险非复发死亡；**C** 竞争风险复发；**D** 逆概率加权竞争风险复发 **注** IPTW：逆概率加权，对年龄、造血干细胞移植合并症指数（HCT-CI）评分、骨髓原始细胞比例、供者类型、移植物抗宿主病（GVHD）预防方式、是否桥接治疗、移植物中单个核细胞（MNC）计数以及移植物中CD34^+^细胞计数进行加权；TCI：预处理强度，TCI评分1～2分为TCI-1组、2.5～3.5分为TCI-2组、4～6分为TCI-3组。NRM：非复发死亡率

IPTW后的Fine-Gray竞争风险模型结果显示，与TCI-1组相比，TCI-2组非复发死亡风险无显著差异（*sHR*＝1.376，95％*CI*：0.392～4.832，*P*＝0.618）；而TCI-3组非复发死亡风险显著升高（*sHR*＝6.277，95％*CI*：1.887～20.871，*P*＝0.003）（表4）。在IPTW加权的基础上，进一步采用DR模型，将年龄、HCT-CI评分、骨髓原始细胞比例、供者类型、GVHD预防方案、移植前是否桥接治疗、移植物中MNC计数以及移植物中CD34^+^细胞计数等基线协变量纳入Fine-Gray竞争风险回归模型。结果显示，TCI-3组仍然是非复发死亡的独立危险因素（*sHR*＝5.011，95％*CI*：1.118～22.452，*P*＝0.035），其他协变量未表现出显著效应，提示TCI-3是非复发死亡的独立危险因素这一结论具有较强稳健性（表4）。

**表4:** 影响骨髓增生异常肿瘤（MDS）患者移植后非复发死亡的IPTW-Fine-Gray主效应模型及双重稳健模型

变量	*sHR*（95％ *CI*）	*P*值
IPTW-Fine-Gray主效应模型		
TCI-2	1.376（0.392～4.832）	0.618
TCI-3	6.277（1.887～20.871）	0.003
IPTW-Fine-Gray双重稳健模型		
TCI-2	1.057（0.236～4.738）	0.942
TCI-3	5.011（1.118～22.452）	0.035
年龄≥50岁	1.664（0.740～3.740）	0.218
移植前HCT-CI>2分	1.443（0.566～3.677）	0.443
移植前骨髓原始细胞比例≥5％	1.347（0.612～2.963）	0.459
HID	2.757（0.277～27.465）	0.387
MUD	1.467（0.131～16.410）	0.756
非ATG预防GVHD	1.236（0.300～5.094）	0.769
移植前桥接治疗	1.069（0.478～2.391）	0.872
移植物中MNC计数	1.024（0.962～1.089）	0.455
移植物中CD34^+^细胞计数	1.029（0.898～1.181）	0.678

**注** IPTW-Fine-Gray：逆概率加权的Fine-Gray模型；TCI：预处理强度，TCI评分1～2分为TCI-1组、2.5～3.5分为TCI-2组、4～6分为TCI-3组；HCT-CI：造血干细胞移植合并症指数；HID：半相合供者；MUD：非血缘全合供者；ATG：抗胸腺细胞球蛋白；GVHD：移植物抗宿主病；MNC：单个核细胞

3. TCI对OS的影响：TCI-1、TCI-2和TCI-3组3年OS率分别为70.3％（95％*CI*：56.3％～87.8％）、78.7％（95％*CI*：72.6％～85.3％）和61.2％（95％*CI*：51.5％～72.7％）（*P*＝0.010）（表2）。IPTW后TCI各组移植后3年OS率分别为62.9％（95％ *CI*：40.6％～83.5％）、79.5％（95％ *CI*：73.3％～85.9％）和55.0％（95％ *CI*：42.8％～66.5％）（*P*<0.001）。

4. TCI对复发的影响：不同TCI处理组的患者整体累积复发率（CIR）差异无统计学意义（*P*＝0.704）（图1C）。3年CIR分别为21.5％（95％ *CI*：10.0％～32.9％）、21.5％（95％ *CI*：14.9％～28.1％）和19.8％（95％ *CI*：10.7％～28.8％）（*P*＝0.957）（表2）；在IPTW后，三组间CIR差异仍无统计学意义（*P*＝0.500）（图1D）。

5. 各TCI组非复发死亡对移植失败的贡献率：总体而言，移植后3年非复发死亡对移植失败的贡献率为39.0％。在各TCI分组中，随TCI强度升高，非复发死亡对移植失败的贡献率呈逐步上升趋势。具体而言，TCI-1、TCI-2和TCI-3组在移植后100 d的非复发死亡贡献率分别为16.3％、43.7％和78.6％（*P*＝0.011），180 d分别为30.1％、41.3％和75.0％（*P*<0.001），1年分别为28.5％、36.2％和75.9％（*P*<0.001），3年分别为26.4％、26.3％和57.8％（*P*<0.001）。多状态模型继续分析不同TCI组在移植后3年内的状态占有概率，直观呈现各TCI组中非复发死亡与复发对移植失败的动态贡献模式（图2）。结果显示，随着TCI评分增高，非复发死亡成为移植失败的主要原因，尤其在TCI-3组，非复发死亡占27.1％，提示高强度预处理显著增加了治疗相关死亡风险。

**图2:**
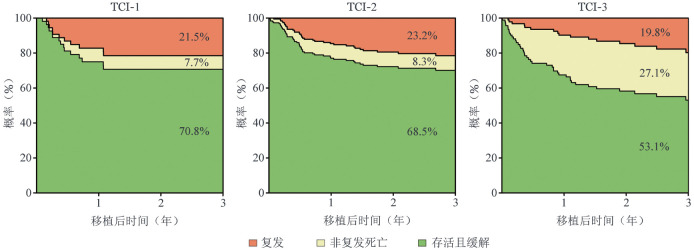
mstate多状态模型分析骨髓增生异常肿瘤不同预处理强度（TCI）分组移植后结局状态概率

## 讨论

本研究基于337例MDS患者的回顾性队列，系统分析了不同TCI对allo-HSCT后非复发死亡、复发及OS的影响。结果显示，TCI-3组显著增加非复发死亡风险，且不降低复发风险，和更差的OS相关，提示高强度预处理可能增加毒性负担，却不能带来额外的抗肿瘤获益。这一发现提示，在MDS人群中，预处理强度的进一步提升可能已超过抗肿瘤效应与治疗毒性之间的最佳平衡点。我们的研究中TCI-3组患者在IPSS-R、IPSS-M分层及移植前骨髓原始细胞比例等疾病负荷指标方面与其他组并无显著差异，且年龄更年轻、HCT-CI评分更低，提示高强度方案的选择主要基于患者生理耐受性而非疾病严重程度。这进一步说明本研究中观察到的NRM升高不太可能单纯由基线疾病负荷差异所解释。进一步采用IPTW及DR模型校正潜在混杂后，TCI-3与NRM升高之间的关联仍保持稳定，支持该结论具有较强因果稳健性。

allo-HSCT是目前唯一能治愈MDS的治疗手段[16]–[17]。预处理强度一般分为MAC和RIC两类，但预处理强度实为连续谱，新药物/组合方案使传统二分法难以准确映射实际生物学剂量。近期，Noureddine等[18]基于EBMT的大样本回顾性研究纳入2014–2018年间≥50岁的1 393例MDS移植患者，其中约75％接受RIC。结果显示，在3年OS、无事件生存（EFS）、复发及非复发死亡方面差异均无统计学意义；IPTW亦未发现能显著从MAC获益的亚组，提示在老年MDS人群中，提高预处理强度并不能带来OS改善，而可能主要增加毒性，进一步强调了个体化强度选择的重要性。Spyridonidis等[9]为常用预处理方案成分分配强度权重分数，并将其总和作为TCI分数。TCI方案对MAC/RIC分类进行了改进，为定义和测量预处理方案强度提供了新工具。进一步研究发现，TCI强度能显著区分移植后早期及两年内的非复发死亡与复发风险，随TCI强度升高，NRM增加而CIR下降。多因素分析进一步证实TCI为两者的独立预测因子，但其对OS的分层作用相对有限，提示预处理强度在复发与毒性之间存在动态平衡[10]。该研究结果表明，TCI相较于传统分法更具连续化和预测价值，为临床决策和方案比较提供了量化工具。合理选择和调整TCI强度，不仅可以提高移植的成功率，还能降低复发风险和移植相关并发症发生率。

RICMAC Ⅲ期随机对照研究的长期随访结果发现，MAC与RIC对MDS患者移植后OS和RFS在总体上无显著差异[19]。然而，在低细胞遗传学风险亚组中，RIC方案表现出更佳的OS、RFS及更低的NRM，而复发风险并未显著增加。这提示对于部分低风险患者，减毒预处理并不劣于甚至可能优于高强度方案，有助于降低治疗相关死亡率，而不牺牲抗白血病效应。TCI评分体系通过量化药物剂量及毒性强度，能更客观地评估预处理强度对患者结局的影响。高TCI评分与NRM增加密切相关，而低中等强度在降低毒性与保持抗白血病效应之间取得了更合理的平衡。

值得注意的是，我们的研究未发现TCI强度与CIR存在显著关联。这提示MDS复发更多的与疾病生物学特征、供受者免疫重建及移植后干预相关，而非单纯依赖预处理强度。因此，对于MDS患者，预处理强度的优化应在降低NRM的前提下，更多结合分子分型、可测量残留病（MRD）水平及移植后维持治疗等综合手段来控制复发风险。最近，北京大学人民医院Wang等[20]发现MDS患者IPSS-R评分和移植后MRD是移植后OS和复发的独立风险因素。

此外，本研究显示年龄与TCI对非复发风险作用无显著交互，说明高强度TCI对非复发死亡的负面效应在不同年龄层患者中一致。然而，老年患者由于基础疾病负担更重，高强度预处理带来的非复发死亡绝对风险上升可能更为显著。因此，在临床决策中，对50岁以上患者尤其应谨慎选择高强度方案。

本研究亦有一定局限性。首先，本研究为多中心回顾性研究，虽采用了IPTW及DR模型以减少混杂，但仍不能完全避免选择偏倚。其次，研究病例数相对有限，部分亚组分析存在统计效能不足。部分中心未提供完整染色体结果，故无法计算IPSS-R评分，导致IPSS-R数据缺失。IPSS-M依赖31个MDS相关基因和预后相关基因突变检测，本研究中部分早期病例（尤其是2019–2020年）未进行系统化二代测序（NGS）检测或基因Panel中未包含评分所需基因突变检测；各中心基因检测平台差异较大，导致部分患者的突变谱无法满足IPSS-M建模要求。同时，本研究未系统收集移植后并发症，如血流感染、侵袭性真菌病和病毒感染的详细资料，无法对不同感染类型进行更精确比较，因此无法系统评估不同TCI对感染类型的具体影响。最后，本研究未能深入分析不同药物组合在相同TCI评分下的差异作用，未来需进一步扩大样本并结合分子生物学数据，以明确不同TCI方案在不同分子亚型MDS中的适应性。

综上所述，本研究表明在MDS allo-HSCT中，过高的预处理强度（TCI-3）显著增加非复发死亡风险，并导致OS率下降，且未能降低复发风险。低中等强度预处理可能在疗效与安全性之间达到最佳平衡。个体化选择预处理强度应综合考虑患者年龄、基础疾病负担、疾病状态及分子风险分层，从而优化移植预后。
